# Anthrax Toxin Receptor 1 Is Essential for Arteriogenesis in a Mouse Model of Hindlimb Ischemia

**DOI:** 10.1371/journal.pone.0146586

**Published:** 2016-01-19

**Authors:** N. J. Andersen, E. A. Boguslawski, A. S. Naidu, C. Szot, J. L. Bromberg-White, K. Kits, C. Y. Kuk, L. E. Holton, B. St. Croix, C. M. Chambers, N. S. Duesbery

**Affiliations:** 1 Frederik Meijer Heart and Vascular Institute, Spectrum Hospital, 100 Michigan St. NE, Grand Rapids, MI, 49503, United States of America; 2 Laboratory of Cancer and Developmental Cell Biology, Center for Cancer Cell Biology, Van Andel Research Institute, 333 Bostwick Ave. NE, Grand Rapids, MI, 49503, United States of America; 3 Tumor Angiogenesis Section, Center for Cancer Research, National Cancer Institute, Bldg. 560, Frederick, MD, 21702, United States of America; 4 Partners in Science Program, Van Andel Education Institute, 333 Bostwick Ave NE, Grand Rapids, MI, 49503, United States of America; Temple University School of Medicine, UNITED STATES

## Abstract

Anthrax toxin receptor 1/tumor endothelial marker 8 (Antxr1 or TEM8) is up-regulated in tumor vasculature and serves as a receptor for anthrax toxin, but its physiologic function is unclear. The objective of this study was to evaluate the role of Antxr1 in arteriogenesis. The role of Antxr1 in arteriogenesis was tested by measuring gene expression and immunohistochemistry in a mouse model of hindlimb ischemia using wild-type and ANTXR1^-/-^ mice. Additional tests were performed by measuring gene expression in *in vitro* models of fluid shear stress and hypoxia, as well as in human muscle tissues obtained from patients having peripheral artery disease. We observed that Antxr1 expression transiently increased in ischemic tissues following femoral artery ligation and that its expression was necessary for arteriogenesis. In the absence of Antxr1, the mean arterial lumen area in ischemic tissues decreased. Antxr1 mRNA and protein expression was positively regulated by fluid shear stress, but not by hypoxia. Furthermore, Antxr1 expression was elevated in human peripheral artery disease requiring lower extremity bypass surgery. These findings demonstrate an essential physiologic role for Antxr1 in arteriogenesis and peripheral artery disease, with important implications for managing ischemia and other arteriogenesis-dependent vascular diseases.

## Introduction

Anthrax toxin receptor 1/tumor endothelial marker 8/(ANTXR1 or TEM8) is a transmembrane protein that is up-regulated in tumor-associated vasculature [1] and can serve as a receptor for the binding moiety of anthrax toxin [2]. The physiologic function of Antxr1 is unclear. Antxr1 appears to play an important role in homeostasis of the extracellular matrix, since *Antxr1*^-/-^ mice show an accumulation of extracellular matrix in several tissues [3]. Studies have shown that the extracellular domain of Antxr1 binds collagens I [4] and VI [5]. Further, evidence of its roles in vascular pathologies including tumor angiogenesis [3], vascular malformations [6], infantile hemangioma [7], and GAPO syndrome [8] suggest it may function in vascular remodeling. Antxr1 expression is not required for non-pathologic angiogenesis [3]. However, the role Antxr1 in arteriogenesis, the radial expansion of existing arteries that is triggered by an increase in fluid shear stress, has not been evaluated. The overall aim of this study was to evaluate the requirement for Antxr1 in arteriogenesis using a mouse model of hindlimb ischemia in which ligation of the femoral artery causes acute loss of perfusion that is restored within 1–2 weeks by both arteriogenesis and angiogenesis [9,10]. Our findings indicate that Antxr1 plays an essential role in arteriogenesis during recovery from femoral artery ligation.

## Materials and Methods

### Ethics Statement

De-identified human tissues were obtained from consenting patients through a protocol approved by the Frederick Meijer Heart and Vascular Institute IRB (#2011–332) and the Van Andel Institute IRB (#14017, 15001). All human subjects provided informed, written consent. These studies meet all of the PLoS Journals’ requirements for research involving human participants. All mouse experiments were done in compliance with the guiding principles of the ‘‘Guide for the Care and Use of Laboratory Animals.” using protocols (2014–0506, 2015–0097) approved by the Van Andel Institute (VAI) Institutional Animal Care and Use Committee (IACUC).

### Cell Culture

All cells were maintained in a humidified 37°C incubator with 5% CO2 at room air (21% oxygen) unless described otherwise. HIAEC (cAP-0020) were obtained from Angio-Proteomie (Boston, MA) and cultured in Endothelial Growth Medium (cAP-02) as described by the manufacturer.

### Fluid Shear Stress and Hypoxia

Fluid shear stress experiments were performed with the ibidi Pump System and PumpControl Software v. 1.5.2 (ibidi USA Inc, Madison, WI). Cells were cultured in flow chambers (μSlide I 0.6 Luer ibiTreat #80186) at the density and incubation times recommended by the manufacturer. Fluid shear stress experiments were automated using the PumpControl Software set to 10 dyn/cm^2^. Flow chambers were incubated at times from 5 min to 24h, after which the cells were collected from the chambers with TRIzol (Ambion) and RNA extracted using the RNeasy kit (Qiagen).

Hypoxia experiments were performed using a hypoxia control system (PLAS Labs). Three independent chambers were used to control oxygen concentrations via attached oxygen sensors to adjust the nitrogen/oxygen gas balance as specified. Each humidified chamber was maintained at 37°C incubator with 5% CO2. For Q-PCR, cells were seeded at 30% confluence in 35mm cell culture dishes for 24h in a 37°C incubator with 5% CO2 and room air before being moved to designated hypoxia chambers. Media was replaced with fresh medium that was preincubated overnight in the hypoxia chambers to equilibrate oxygen concentrations. Cells were incubated in respective hypoxia chambers for 72 hr prior to RNA collection.

### Murine Hindlimb Ischemia Model

All mouse experiments were done in compliance with the guiding principles of the ‘‘Guide for the Care and Use of Laboratory Animals.” using protocols (2014–0506, 2015–0097) approved by the Van Andel Institute (VAI) Institutional Animal Care and Use Committee (IACUC). Athymic NCr-nu/nu and C57Bl/6 mice were bred and housed in an AAALAC-accredited specific-pathogen-free environment with a 12 hour light-dark cycle and provided food and water *ad libidum*. *Antxr1*^-/-^ mice [3] were bred at the National Cancer Institute in Frederick, Maryland and transported to Van Andel Research Institute. These mice TEM8 KO mice were originally made on a mixed 129SvJae/C57BL6 genetic background and have been backcrossed >10 generations onto a C57BL6 background. Experiments were carried out under an IACUC-approved protocol and institutional guidelines for the proper and humane use of animals in research were followed. Femoral artery ligation was performed on the right leg as described by Couffinhal et al. [9]. Mice were anesthetized using 2% Isoflurane by inhalation. A subcutaneous injection of ketoprofen at 5mg/kg was given prior to surgery as an analgesic. Two sutures were placed proximal and distal to the superficial caudal epigastric artery [11]. A third suture was placed over both the superficial caudal epigastric artery and the proximal caudal femoral artery and the intervening segment was excised. Some mice received sham surgery, which was identical in all aspects to femoral artery ligation except that the artery and its branches were neither ligated nor excised. FP59 and PA were formulated in PBS. Seven days after femoral artery ligation, equal doses (0.012 mg/ml) of PA and FP59 were administered i.p. in a 100 μl total volume. After 24 h, mice were euthanized by retro-orbital exsanguination followed by cervical dislocation. Each calf was removed and dissected longitudinally for formalin fixed paraffin embedding and snap fresh freezing. All animal procedures were performed with the experimenter blinded to the identity of the mouse or the nature of its treatment.

### Antibodies and Immunostaining

Mice were euthanized at various time points post surgery. Leg tissues were surgically removed and formalin-fixed. Calf tissues were first decalcified with ImmunoCal decalcifying solution (Decal Chemical Corporation, Tallman NY) and then were embedded in paraffin. In some experiments calves were segmented into thirds prior to decalcification and embedding in paraffin. Immunohistochemical (IHC) staining was performed with the use of optimized standard protocols on an automated stainer (Discovery XT; Ventana Medical Systems, Tucson AZ) using anti-CD31/PECAM-1 (Lab Vision, Kalamazoo, MI; 1:100) antibodies. Horseradish peroxidase–conjugated anti-rabbit IgG (Ventana Medical Systems UltraMap; 1:100) or anti-rat IgG (Ventana Medical Systems UltraMap; 1:100) were used as a secondary antibody, and slides were developed with 3–3’-diaminobenzidine chromogen substrate. For Antxr1 staining, deparaffinized tissues were treated with proteinase K (Dako) and blocked with Dual Endogenous Enzyme-Blocking Reagent followed by a Biotin-Blocking System (Dako). Sections were incubated with a rabbit monoclonal antibody against Antxr1 (clone 37) and detected using the Vectastain Elite ABC Kit (rabbit IgG) from Vector Labs. This rabbit monoclonal antibody was produced as part of a collaboration between Epitomics (AbCam) and the National Cancer Institute and will be described in more detail elsewhere.

Hematoxylin and eosin (basophilic) staining was performed using the automated Symphony system (Ventana Medical Systems). Because of batch-to-batch variability in staining intensity, wherever possible all slides for a single experiment were processed at the same time. IHC and basophilic staining were quantified by multispectral imaging using a Nuance Scanscope (Aperio, Vista CA). Quantification was performed with the researcher being blinded to the identities of the samples being measured. For IHC, sections were obtained from the lower, mid, and upper calf muscle. Three images were quantified per section. The nine measurements thus obtained were averaged to obtain a single value for the entire limb. Then, the values for each limb (n = 3–6) were averaged to obtain the experimental results and standard deviation. In separate blinded analyses, the average arterial cross-sectional area was calculated by measuring the 3 largest arteries per section using Spot imaging software (Diagnostic Instruments, Sterling Heights, MI). As with IHC, the nine measurements obtained for each leg were averaged to obtain a single value for the entire limb. Then, the values for each limb (n = 3–6) were averaged to obtain the experimental results and standard deviation. Direct measurement of arterial lumen size was necessary since the image resolution achievable with contrast angiography is on the order of 0.2–0.3 mm.

### Immunoblotting

Muscle tissues were lysed in TNT lysis buffer (50 mM Tris [pH 7.5], 75 mM NaCl, and 1% Triton X-100 plus complete protease inhibitor cocktail [Roche]) and clarified by centrifugation. Protein extracts were quantified with a BCA assay (Pierce), normalized, separated by SDS PAGE, transferred to a polyvinylidene difluoride membrane (Millipore) and blotted using a rabbit monoclonal ANTXR1 antibody (clone 37), followed by an HRP-anti-rabbit secondary (Jackson Immunoresearch). Proteins were visualized using the enhanced chemiluminescence plus system (Amersham) according to the supplier’s instructions.

### Quantitative PCR

Snap frozen fresh tissue was homogenized in Trizol (Life Technologies) using a TissueLyzer II (Qiagen) at 30 Hz for 2 min. Chloroform was added and the supernatant was centrifuged at 4C. 70% ispropanol was added to the aqueous phase and then passed through an RNeasy spin column (Qiagen). The column was washed with RNeasy RWI buffer, treated with RNASe-free DNAse (Qiagen), washed with RNeasy RWI buffer, and RPE (Qiagen). RNA was eluted in water. cDNA was generated from RNA using a High Capacity cDNA Reverse Transcription kit (Applied Biosystems) following the manufacturer’s instructions.

Power SYBR Green PCR Master Mix (Applied Biosystems) was used for qPCR following manufacturer’s protocol using a 7500 Real Time PCR machine (Applied Biosystems). For Antxr1 and Antxr2 expression in human surgical samples, the mean quantity was calculated using a standard curve. For each sample, the mean quantity of Antxr1 and Antxr2 were normalized to the mean quantity of 18S. The average and standard deviation were calculated for each patient population. For *in vitro* and mouse studies, Antxr1 and Antxr2 C_t_ were subtracted from 18S to calculate the △C_t_. The expression was normalized to expression at day 0 (2^(△C_t_ (treated)-△C_t_(0 h)). The average normalized gene expression and the standard deviation were calculated. *In vitro* expression data represents by three to four independent experiments. Standard qPCR conditions were 50 C for 2 m, 95 C for 10 m, followed by 40 cycles of 95 C for 15 s and 60 C for 1 min. Dissociation analysis was performed to verify that the samples contained a single product. The following primers were used: Antxr1 (human forward 5’-tgctgcaccactggaatgaaatc-3’, reverse 5’-ctcctcctggcagaactttctgg-3’; mouse forward 5’-atggcccacagtagatgcc-3’, reverse 5’-gaagttgatacagcgtccgg-3’), Antxr2 (human forward 5’-ctttcattgtgttttcttctcaagcaac-3’, reverse 5’-gttttcaagcctcctgctyyctgaat-3’; mouse forward 5’-ctcttgcaaaaaagccttcg-3’, reverse 5’-ttctttgcctcgttctctgc-3’), and 18S (forward 5’-gtaacccgttgaaccccatt-3’, reverse 5’-ccatccaatcggtagtagcg-3’) [3,12,13].

### Statistics

All data are plotted as the mean of at least 3 independent experiments ± standard deviation. Statistical significance was determined using a two-tailed *t* test, where *P* ≤ 0.05 was considered significant. All the data described in this manuscript are freely available at Dryad doi:10.5061/dryad.b8d0g.

## Results

**Fig 1:**
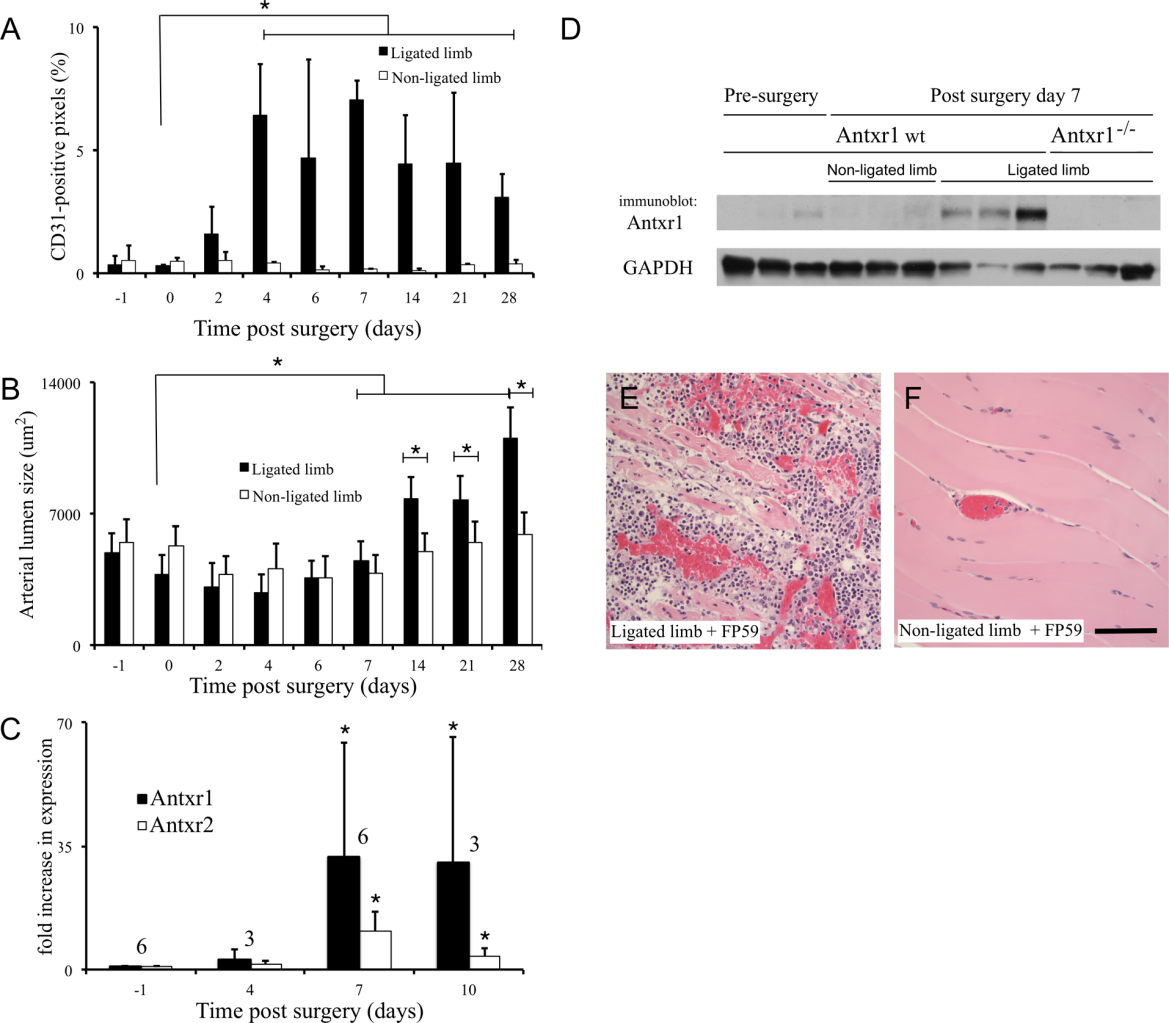
Increased Antxr1 expression precedes arteriogenesis. Increased angiogenesis following femoral artery ligation in athymic NCr-nu/nu mice was measured by IHC with antibodies against CD31 and quantification using Nuance spectral imaging, *n* = 5 mice per group (A). Microscopic measurements of arterial lumen area using Spot software showed a later increase begining 7–14 days post-ligation, *n* = 5 mice per group (B). Quantitative PCR of Antxr1 and Antxr2 revealed a transient elevation of Antxr1 and Antxr2 mRNA around 7 days post surgery, the number of independent replicates is indicated above each pair of bars (C). Immunoblotting with antibodies against Antxr1 indicated protein expression was increased at 7 days post-ligation, *n* = 3 mice per group (D). Hematoxylin and eosin staining showed the injection of PA and cytotoxic FP59 caused vascular injury in the ligated leg (E) but not in the contralateral limb (F). The error bars show standard deviation, the asterisks indicate *P* ≤ 0.05, and the scale bar = 100 μm.

To evaluate endothelial cell response to femoral artery ligation we quantified immunohistochemical (IHC) staining for the endothelial cell marker CD31. Our studies were performed using athymic NCr-nu/nu and C57BL/6 mice. Comparable results were achieved for each strain, though C57BL/6 mice recovered faster. Staining in areas of muscle regeneration showed a rapid angiogenic growth of microcapillaries 2–4 days post-ligation (Fig 1A, S1 Fig) that was followed by growth in arterial lumen area in peripheral regions and between muscle fibers beginning between 7 and 14 days (Fig 1B, S1 Fig). Quantitative RT-PCR (qPCR) of muscle tissues revealed that Antxr1 mRNA expression increased 20-fold between 4 and 7 days following femoral artery ligation, prior to the onset of arteriogenesis (Fig 1C, S1 Fig). A similar, though lesser increase in the expression level of the related anthrax toxin receptor 2 was also measured. These results were confirmed by immunoprecipitation and immunoblotting (Fig 1D) and immunohistochemistry (S2 Fig), the latter revealing expression in bth vascular and perivascular stromal cells. Antxr1 expressing cells can be targeted with protective antigen (PA) and FP59, a recombinant fusion toxin consisting of anthrax lethal factor (LF) amino acids 1–254 (LFn) fused to the ADP-ribosylation domain of Pseudomonas exotoxin A [14]. A pathologic evaluation of muscle tissue from mice treated with an intravenous injection of PA and FP59 seven days after femoral artery ligation showed clear evidence of vascular injury including focal to widespread hemorrhage in the surgical leg (Fig 1E) but not in the contralateral leg (Fig 1F). These data show that Antxr1 expression was increased in the vasculature and perivascular stroma of the surgical limb following femoral artery ligation.

**Fig 2:**
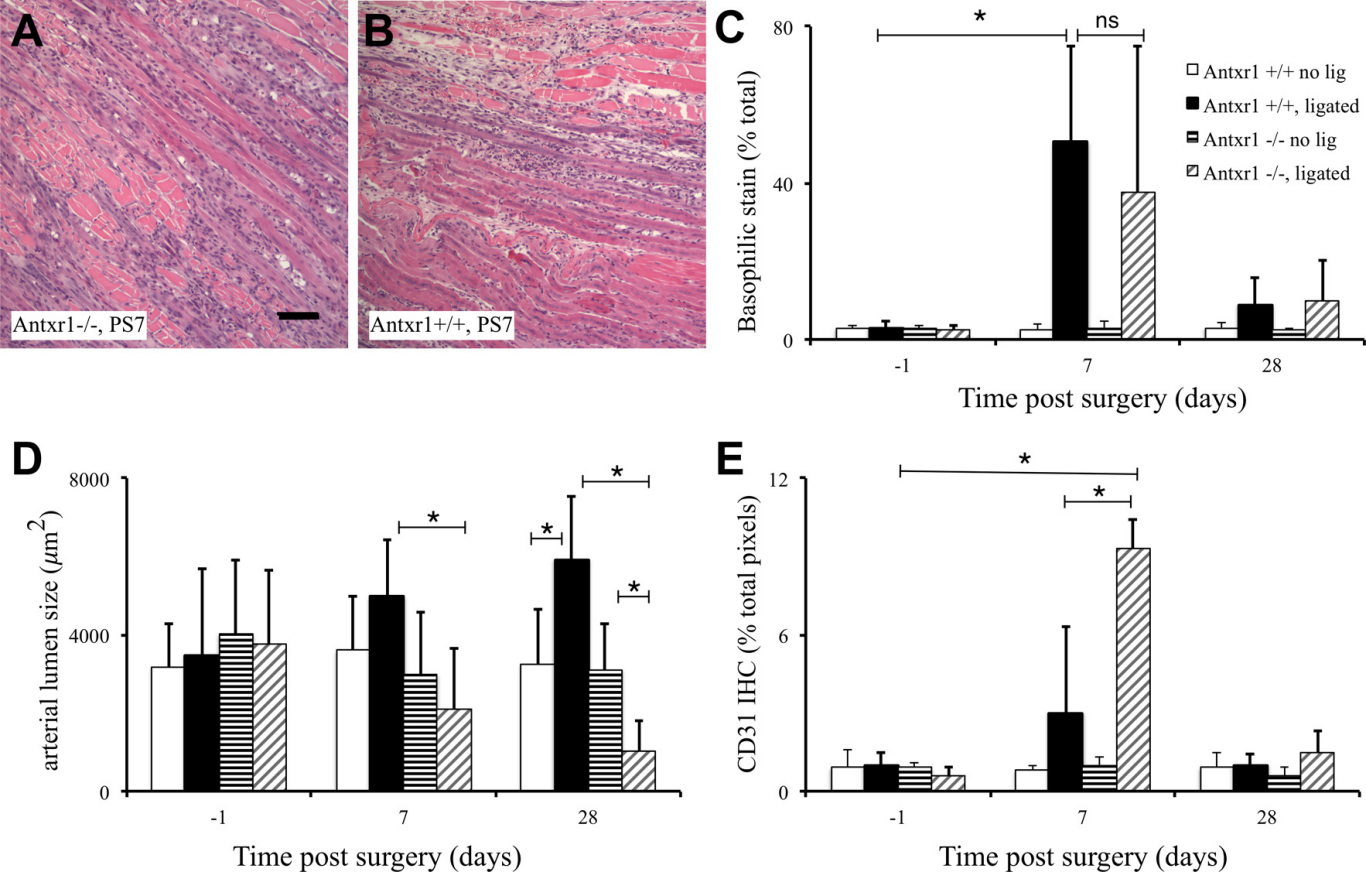
*Antxr1*^*-/-*^ mice recover without arteriogenesis. Hematoxylin and eosin staining revealed no apparent differences in the extent of injury or the ability to regenerate muscle between knock-out mice (A) and wild-type littermates (B) 7 days post surgery. Measuring nuclear density (basophilic stain) by Nuance spectral imaging supported this conclusion (C). Microscopic measurements of arterial lumen area using Spot software showed a decrease in arterial diameter in *Antxr1*^*-/-*^ mice but not in wild-type mice (D). Measurement of angiogenesis by IHC staining with antibodies against CD31 and quantification using Nuance spectral imaging showed an elevated CD31 in *Antxr1*^*-/-*^ compared with wild-type littermates (E). The error bars show standard deviation, *n* = 4 mice per group, the asterisks indicate *P* ≤ 0.05, and the scale bar = 100 μm.

We next used Antxr1 knock-out mice [3] to test the necessity of Antxr1 for recovery from femoral artery ligation. Wild-type littermate mice were used as controls. Hematoxylin-staining (nuclei-dense, basophilic) regions of muscle regeneration, composed of necrotic tissue, infiltrating immune cells, proliferating myocytes and neovascular structures, formed in the surgical leg but not the contralateral leg following femoral artery ligation. The extent of basophilic staining at 7 and 28 days post surgery showed no differences in the extent of injury or the ability to regenerate muscle between knock-out and wild-type mice (Fig 2A–2C). However, whereas the average arterial lumen area in wild-type mice increased between 7 and 28 days post-surgery, mean arterial lumen area in Antxr1 knock-out mice failed to increase in diameter from 7 days onwards, and at 28 days post-surgery was significantly smaller than arteries in the contralateral limb (Fig 2D). In comparison to wild-type mice, Antxr1 knock-out mice showed increased immunohistochemical staining for CD31+ that was associated with capillaries at day 7 (Fig 2E). Thus, our data indicate it is possible to experimentally separate arteriogenesis and angiogenesis in this model and they provide evidence that Antxr1 expression is necessary for the former but not the latter. In addition, our data indicate that Antxr1 is required for the increase in arterial lumen size and that in the absence of arteriogenesis, reperfusion is achieved through a compensatory increase in angiogenesis.

**Fig 3:**
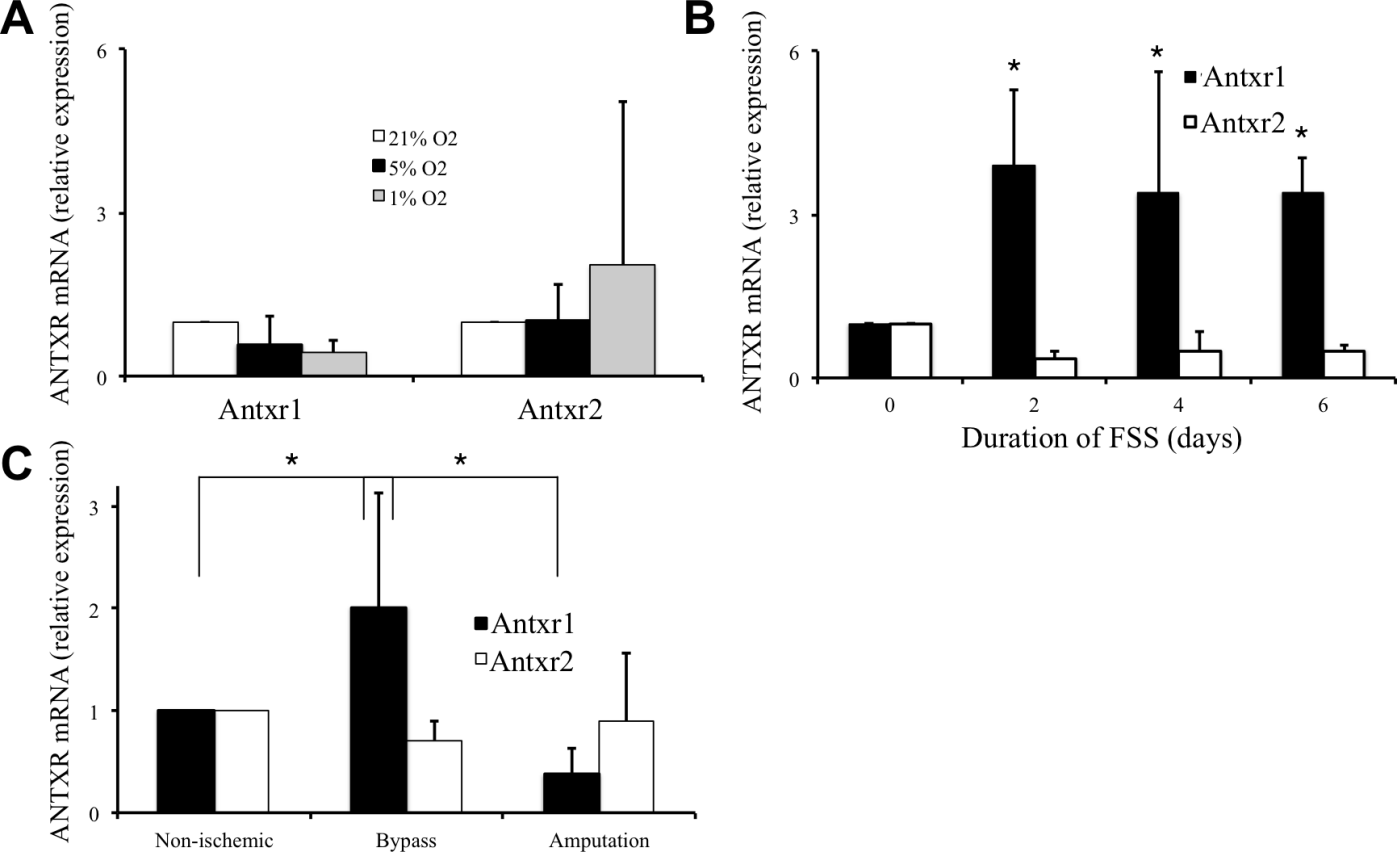
Decreases in oxygen induced Antxr1 transcription and expression at the cell surface. Quantitative PCR of Antxr1 and Antxr2 mRNA from human primary endothelial cells grown at 21%, 5% or 1% O_2_ for 3 days, *n =* 4 independent replicates per condition (A) or subjected to fluid shear stress for 2, 4, or 6 days, *n =* 3 independent replicates per condition (B). Data are normalized to expression at 21% O_2_ or 0 days, respectively. Quantitative PCR of Antxr1 and Antxr2 mRNA in calf muscle samples from 22 healthy patients, 5 chronically ischemic patients receiving a lower extremity bypass surgery, and 5 chronically ischemic patients having a major limb amputation (C). For all graphs, the error bars show standard deviation and the asterisks indicate *P* ≤ 0.05.

Whereas angiogenesis is driven by hypoxia [15,16], arteriogenesis is triggered by increased fluid shear stress[17]. To determine how Antxr1 expression is regulated, we first exposed human iliac artery endothelial cells (HIAEC) to ambient oxygen (21%), normoxic (5% O_2_), or hypoxic (1% O_2_) growth conditions for 24–72 h. We were unable to detect significant changes in either Antxr1 or Antxr2 mRNA levels by qPCR (Fig 3A). However, exposure of HIAEC to 10 dyn/cm^2^ shear induced a significant 4-fold up-regulation of Antxr1 mRNA within 48h that was sustained for up to 6 days (Fig 3B). In contrast, Antxr2 levels were reduced over the same period of observation. These data show that the increase in Antxr1 expression in iliac artery endothelial cells is caused by fluid shear stress that is associated with arteriogenesis but not by hypoxia that drives angiogenesis.

In humans, increases in fluid shear stress induce arteriogenesis that plays a crucial role in the expansion of collateral arteries as a physiologic response to arterial occlusion. However, patients with peripheral arterial disease (PAD) show diminished arteriogenesis, leading to progressive ischemia and limb necrosis. The reasons why some patients recover from arterial occlusion while others develop PAD are not well understood. To determine whether Antxr1 may contribute to the pathogenesis of human disease, we evaluated Antxr1 expression levels in muscle tissues from patients diagnosed with peripheral artery disease. We observed that compared with normal calf muscle, Antxr1 expression in calf muscle obtained from patients receiving a lower extremity bypass to restore blood flow was significantly elevated (Fig 3C). However, Antxr1 expression in calf muscle from patients with advanced disease requiring major limb amputation was significantly lower. In contrast, Antxr2 levels were similar in all samples (Fig 3C). These observations indicate that Antxr1 expression is altered in patients with peripheral arterial disease.

## Discussion

The purpose of this study was to evaluate the requirement for Antxr1 in arteriogenesis using a mouse model of hindlimb ischemia. Our finding indicate that Antxr1 expression increases in the vasculature and perivascular stroma of limbs following ligation of the femoral artery. This increase is necessary for arteriogenesis to occur but not for angiogenesis. Consistent with this, we observed that Antxr1 expression in iliac artery endothelial cells is caused by fluid shear stress that is associated with arteriogenesis, but not by hypoxia that drives angiogenesis. Interestingly, we found that Antxr1 is required for increases in arterial lumen size and that in the absence of arteriogenesis, reperfusion may be achieved through a compensatory increase in angiogenesis. Therefore, we conclude that expression of Antxr1 is critical for arteriogenesis that is induced following an acute loss of perfusion to the hindlimb.

Our study establishes a new physiologic role for this anthrax toxin receptor. Exactly how antxr1 contributes to arteriogenesis is unclear. The related anthrax toxin receptor Antxr2 is reported to be a collagen receptor that is essential for maintaining collagen homeostasis in the uterus [18]. It is possible that Antxr1 plays a similar role, binding and remodeling the basement membrane surrounding blood vessels. Indeed, *Antxr1*^-/-^ mice show an accumulation of extracellular matrix in several tissues [3], suggesting Antxr1 is important for homeostasis of the extracellular matrix.

Antxr1-mediated vascular remodeling may play an important role in the pathogenesis of anthrax. Rates of endothelial cell proliferation and the size of blood vessels increases rapidly after the onset of infection that leads to chronic inflammatory disease [19–22]. These remodeled capillaries and venules are sites of local plasma leakage and leukocyte adherence [23]. By targeting cells involved in vascular remodeling, *Bacillus anthracis* may take advantage of this leakiness to establish a systemic infection. In this regard, it would be interesting to determine whether Antxr deficient mice are more resistant to infection from inhaled anthrax spores.

Our results also raise interesting questions on the role of arteriogenesis in tumor biology. Previous studies have demonstrated that targeting Antxr1 reduces tumor vascular density and is an effective strategy to slow tumor growth and prolong survival [24–29]. Our data indicate that the success of this strategy may be due in part to the necessity of Antxr1 for arteriogenesis. Although a role for arteriogenesis in tumor growth is not widely appreciated, some reports have described the radial expansion of arteries that feed growing tumors [30,31]. Targeting extratumoral arteriogenesis may therefore be an effective strategy to inhibit tumor growth.

Finally, our observation that altered levels of Antxr1 are associated with peripheral artery disease in humans may open the door to development of tests to identify individuals at risk for progressive disease and in greater need of medical intervention. Several splice variants of Antxr1, as well as disease-associated mutations, have been identified and may contribute to disease risk or progression. Further characterization of the role of Antxr1 in arteriogenesis will be an important step in the development of prognostic markers, diagnostic markers, and therapies for the treatment of vascular disease.

## Conclusions

Using a mouse model of hindlimb ischemia, we demonstrate that increased expression of anthrax toxin receptor 1 (Antxr1) is necessary for arteriogenesis, the radial expansion of existing arteries. These results are important, because arteriogenesis plays a crucial role in the expansion of collateral arteries as a physiologic response to arterial occlusion. Patients with peripheral arterial disease (PAD) show diminished expansion of collateral arteries, leading to progressive hypoxia and limb necrosis. Consistent with this, Andersen *et al*. found that altered expression of Antxr1 was associated with peripheral artery disease.

## Supporting Information

S1 Fig Angiogenesis and arteriogenesis in C57Bl/6 mice. Increased angiogenesis following femoral artery ligation was measured by IHC staining with antibodies against CD31 and quantified using Nuance spectral imaging, *n =* 4 mice per group (**A**). Microscopic measurements of artery lumenal area using Spot software showed a later increase beginning >7 days post-ligation, *n =* 4 mice per group (**B**). Quantitative PCR of Antxr1 and Antxr2 revealed a transient elevation of Antxr1 and Antxr2 mRNA at 2 days post surgery, the number of independent replicates is indicated above each pair of bars (**C**).
(DOCX)

S2 Fig Antxr1 staining in ischemic legs. Antxr1 expression was readily detectable in ischemic muscle stroma 7 days post-ligation in Antxr1 wild-type mice, but was undetectable in the non-ligated contralateral muscle, or in ligated muscle from Antxr1 knockout mice. Some non-specific RBC staining can be seen in the control vessels. TEM8 KO mice were originally made on a mixed 129SvJae/C57BL6 genetic background and have been backcrossed >10 generations onto a C57BL6 background. Wild-type littermate mice were used as controls.
(DOCX)
