# Supplementary figures and images for: Anthrax Toxin Receptor 1 Is Essential for Arteriogenesis in a Mouse Model of Hindlimb Ischemia
Source: PLoS One. 2016 Jan 19;11(1):e0146586. doi: 10.1371/journal.pone.0146586 (PMC4718698; doi:10.1371/journal.pone.0146586)

S1 Fig. Angiogenesis and arteriogenesis in C57Bl/6 mice.
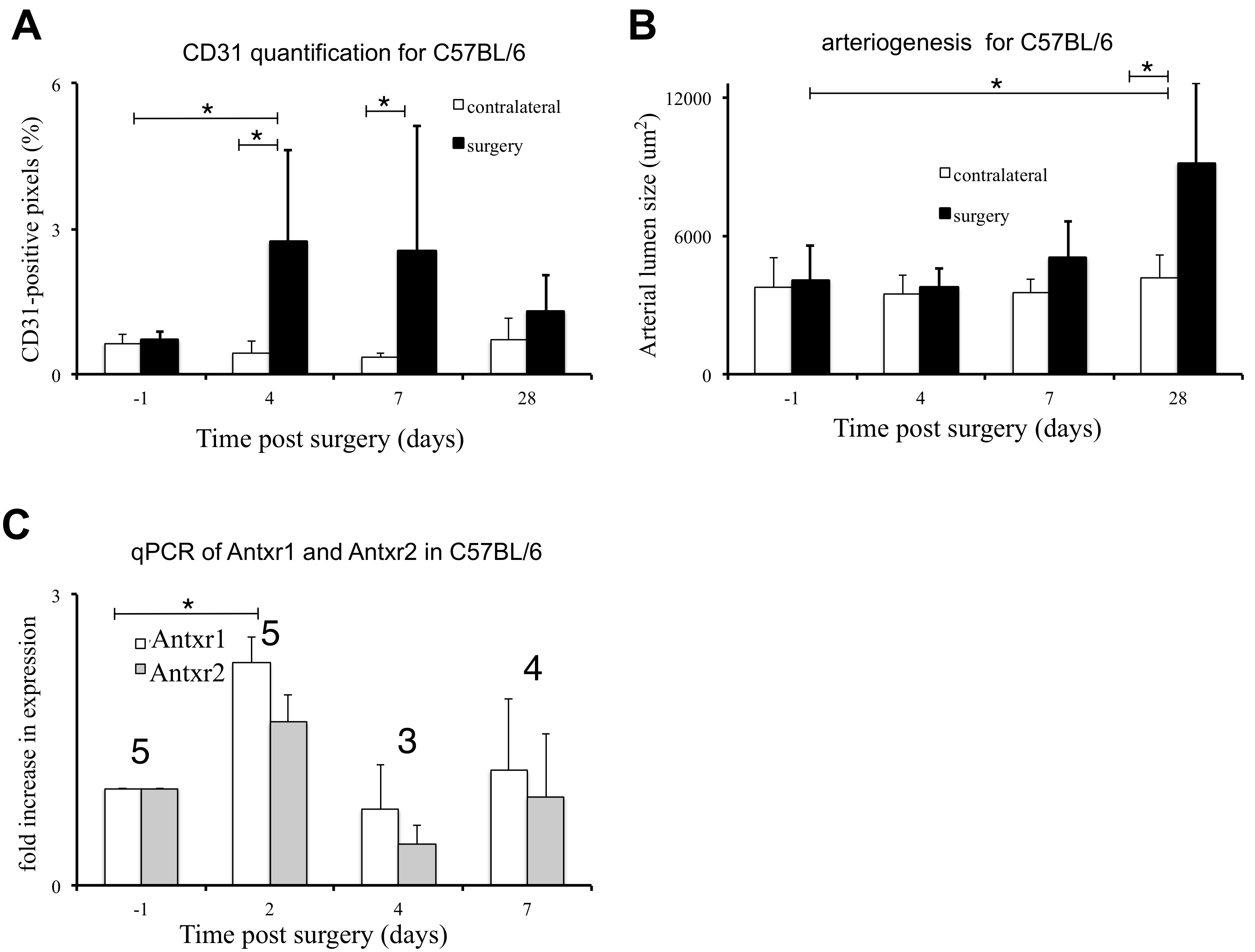

Supplement: S1 Fig — Increased angiogenesis following femoral artery ligation was measured by IHC staining with antibodies against CD31 and quantified using Nuance spectral imaging, n = 4 mice per group (A). Microscopic measurements of artery lumenal area using Spot software showed a later increase beginning >7 days post-ligation, n = 4 mice per group (B). Quantitative PCR of Antxr1 and Antxr2 revealed a transient elevation of Antxr1 and Antxr2 mRNA at 2 days post surgery, the number of independent replicates is indicated above each pair of bars (C). (DOCX) [file pone.0146586.s001.docx]

**S2 Fig. Antxr1 staining in ischemic legs.**
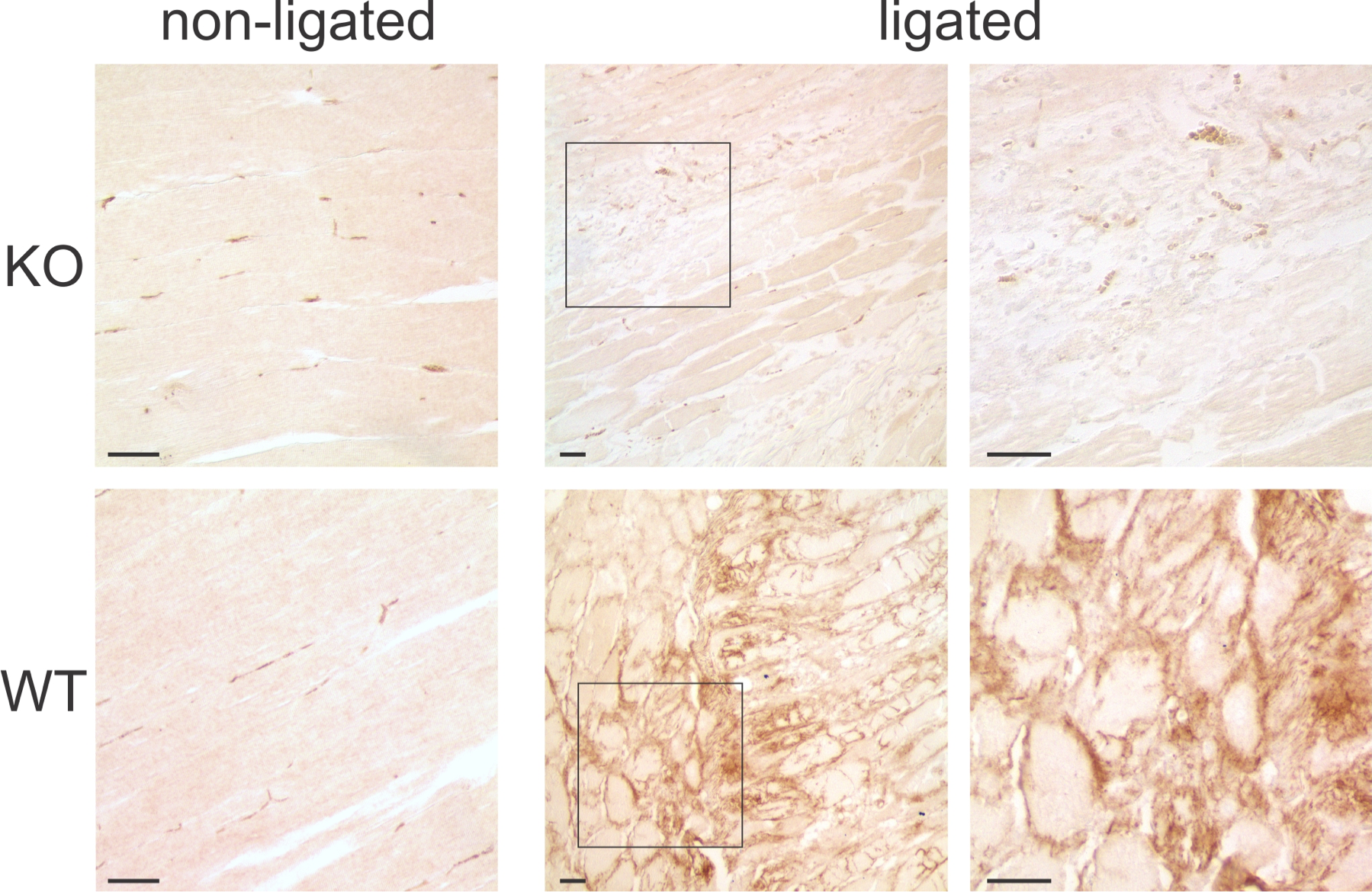

Supplement: S2 Fig — Antxr1 expression was readily detectable in ischemic muscle stroma 7 days post-ligation in Antxr1 wild-type mice, but was undetectable in the non-ligated contralateral muscle, or in ligated muscle from Antxr1 knockout mice. Some non-specific RBC staining can be seen in the control vessels. TEM8 KO mice were originally made on a mixed 129SvJae/C57BL6 genetic background and have been backcrossed >10 generations onto a C57BL6 background. Wild-type littermate mice were used as controls. (DOCX) [file pone.0146586.s002.docx]
